# Esophageal perforation more than one year following anterior cervical spinal surgery

**DOI:** 10.1093/jscr/rjaf399

**Published:** 2025-06-13

**Authors:** Grace R Fassina, Nangorgo J Coulibaly, Griffin L Ernst, Jose A Sanclement, Caple A Spence

**Affiliations:** Department of Neurosurgery, University of Oklahoma, 1000 N Lincoln Blvd #400, Oklahoma City, OK 73104, United States; Department of Neurosurgery, University of Oklahoma, 1000 N Lincoln Blvd #400, Oklahoma City, OK 73104, United States; Department of Neurosurgery, Rush University Medical Center, 1520 W Harrison St., Chicago, IL 60612, United States; Department of Otolaryngology, University of Oklahoma, 800 Stanton L Young Blvd, Oklahoma City, OK 73117, United States; Department of Neurosurgery, Integris Health, 3343 NW 56th St. Suite 900, Oklahoma City, OK 73112, United States

**Keywords:** anterior, cervical, spinal surgery, delayed, esophageal perforation

## Abstract

We illustrate a rare case of a delayed esophageal perforation occurring ˃1 year after anterior cervical spinal surgery for trauma, without evidence of instrumentation failure. The patient presented with dysphagia, headache, and posterior neck pain. A swallow study confirmed posterior esophageal defect and surgical repair was performed using pectoralis flap. Literature review revealed limited cases of esophageal perforation beyond one year later following anterior cervical spinal surgery related to trauma without hardware failure. Most delayed esophageal injuries occur within 2 years and are related to instrumentation failure; cases without instrumentation failure are not commonly presented. Despite their rarity, clinicians should consider esophageal perforation in patients with a prior history of anterior cervical spinal surgery presenting symptoms of esophageal dysfunction. This case report emphasizes prompt recognition and intervention are essential for better outcomes; recommending multidisciplinary approach with neurological surgery and otolaryngology to perform diagnostics and necessary interventions.

## Introduction

Anterior cervical corpectomy or discectomy with instrumented fusion are common procedures performed for various degenerative and traumatic pathologies. Access is typically via the Smith and Robinson approach [1, 2] which involves manipulation of the esophagus to reach the cervical spine. Upon access, the cervical spine is manipulated, portions of the vertebral body may be removed, an interspace/interbody graft is placed, then followed by anterior plating [2]. Complications include carotid compression, airway issues, dysphagia, esophageal perforations, implant failure, or anterior graft migration causing abnormal spinal curvature [3]. Esophageal perforation are potentially life threatening as secretions and bacteria can enter the mediastinum. Mortality rates are as high as 50% [4]. Though the true incidence of esophageal perforations following anterior cervical spine surgery remains unclear due to infrequent reporting; it is estimated to be 0.25% –1.49% [5] with a mortality rate between 6% and 34% [6]. A systematic review by Halani *et al*. [7] identified 46 delayed cases (>30 days), many involving hardware-related complications with a few involving intact hardware. This case report highlights a rare delayed esophageal perforation over one year after apparently successful anterior cervical surgery related to traumatic injury, despite intact spinal instrumentation.

## Case report

**Figure 1:**
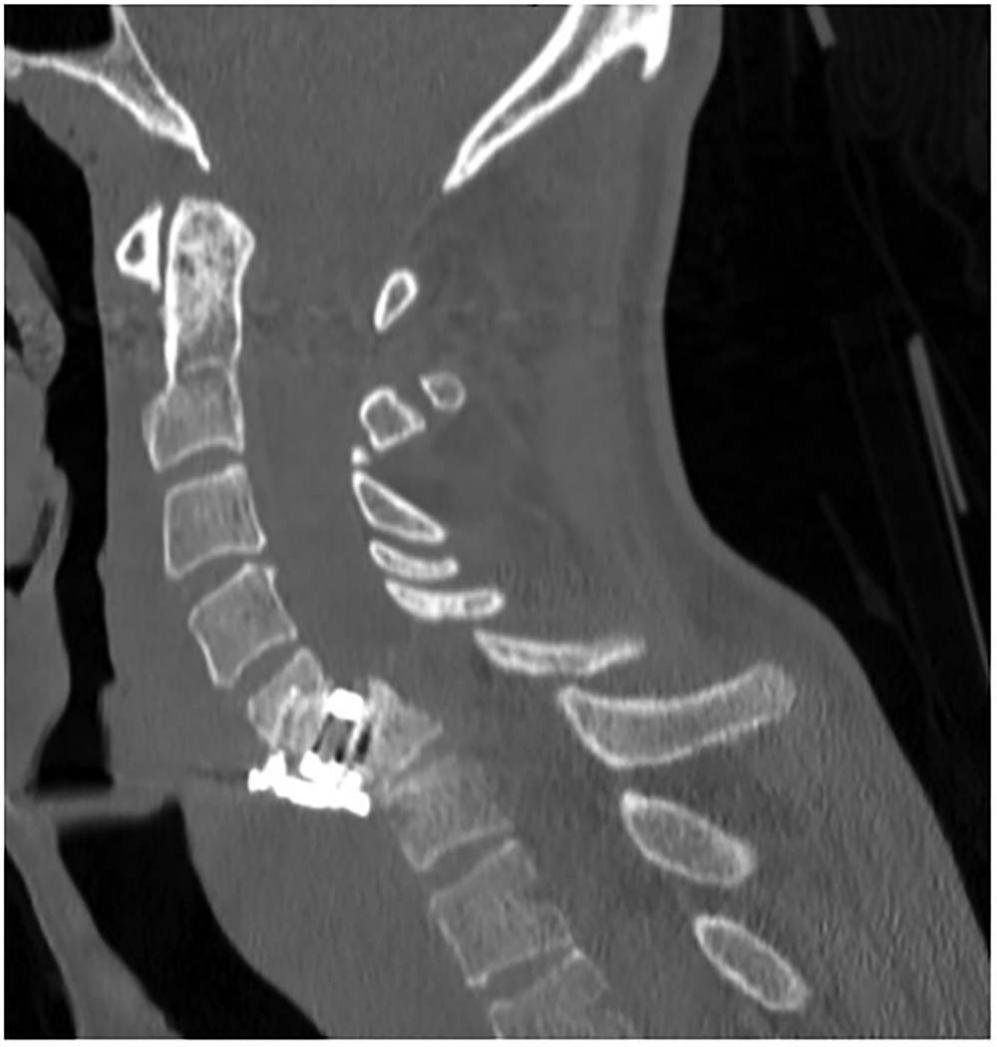
Sagittal CT of initial ACDF at cervical spine C5/C6 level over one year prior to esophageal perforation.

**Figure 2:**
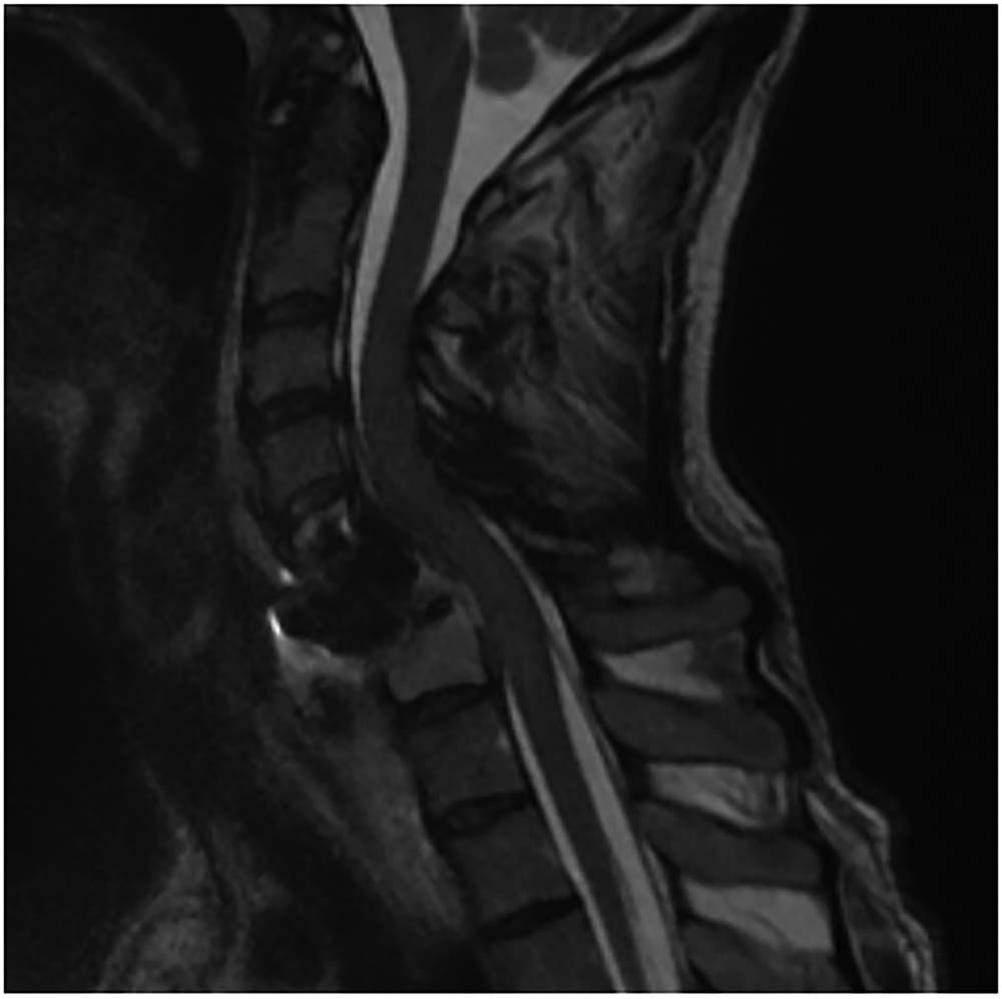
Sagittal T2-weighted MR of prior retropharyngeal abscess that led to revision and posterior plating from C5 to T1.

**Figure 3:**
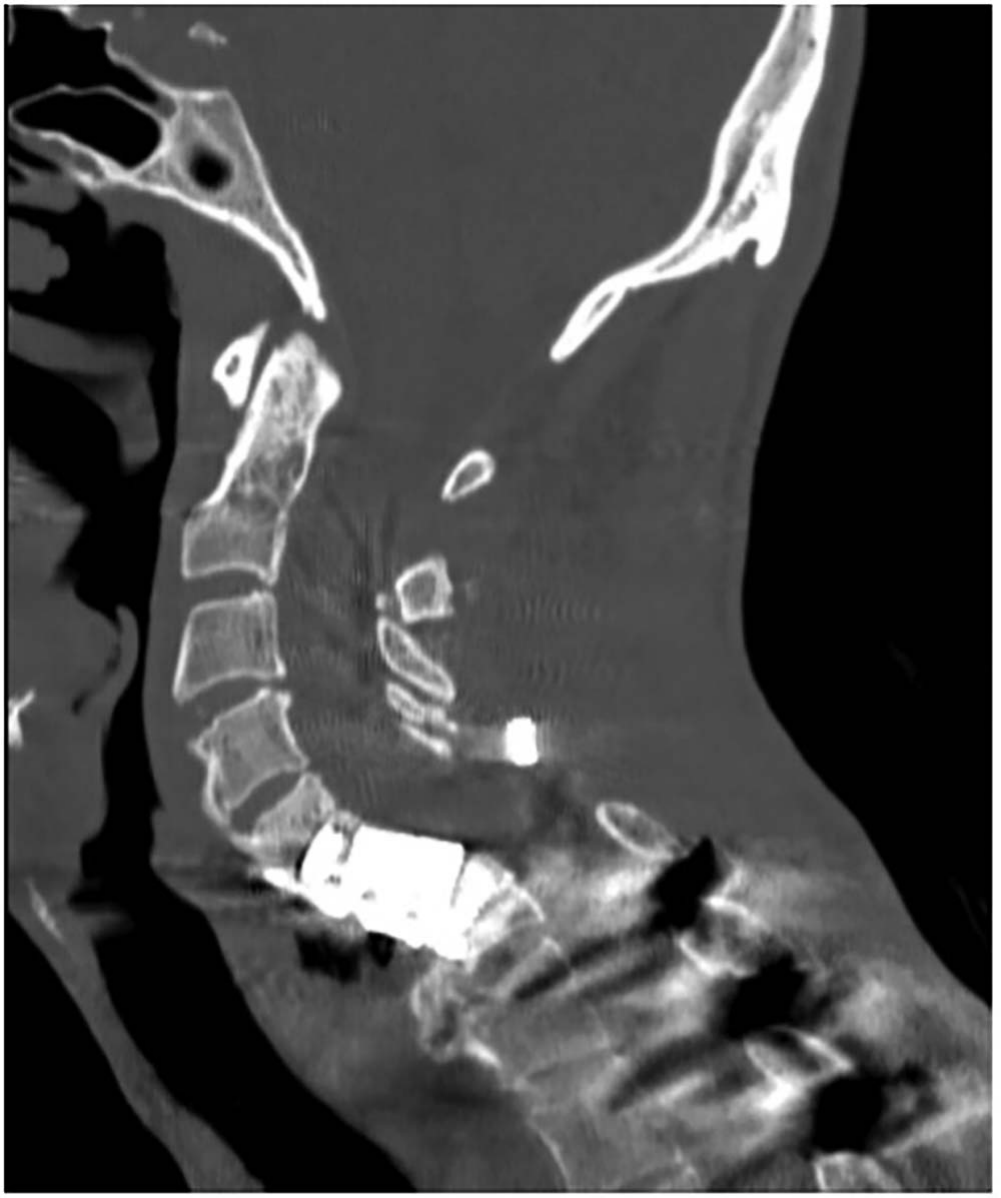
Initial sagittal CT without contrast after patient presented with symptoms of esophageal injury demonstrating increased gas density at the site of C6 corpectomy and post cricoid region.

**Figure 4:**
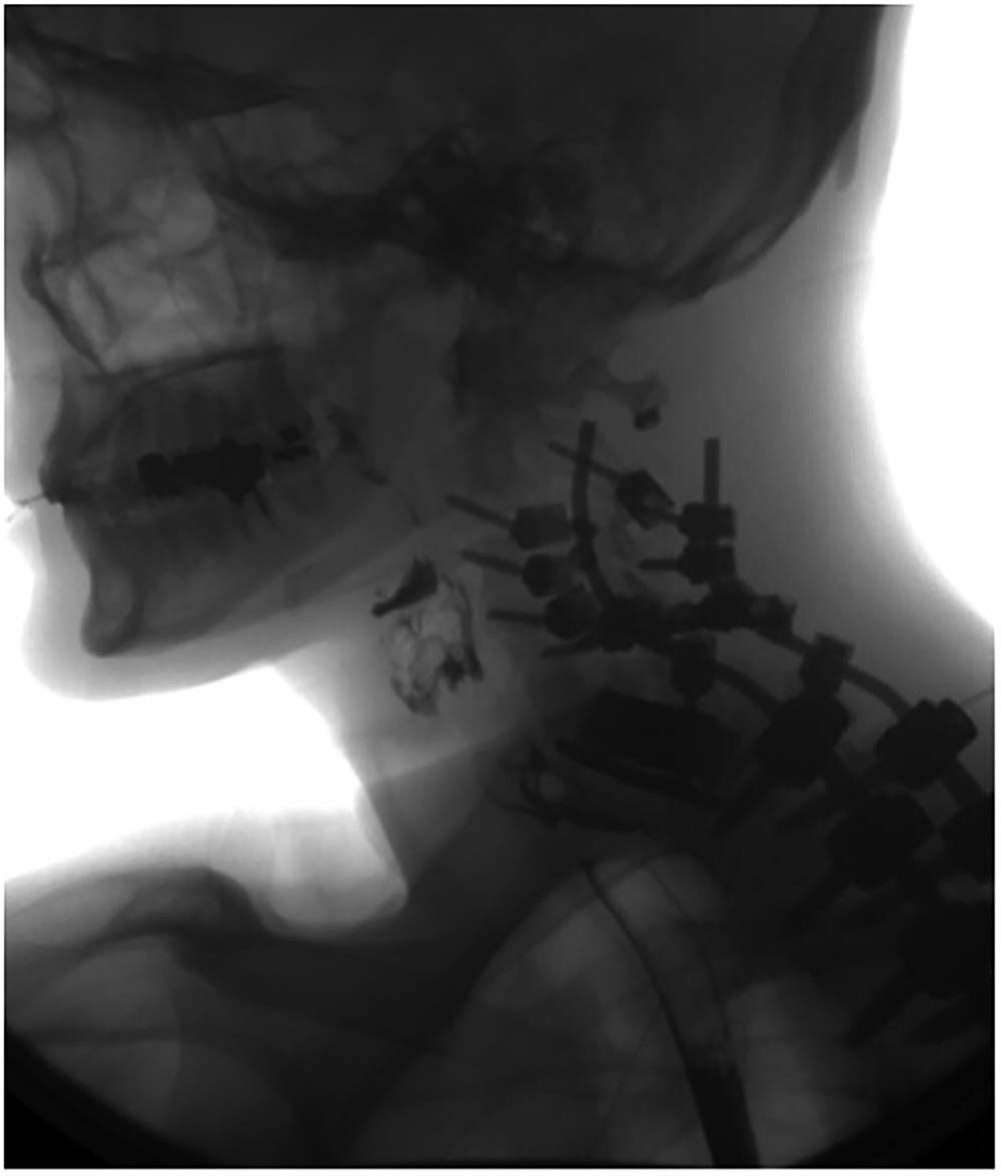
Swallow study demonstrating extraluminal leakage of contrast posteriorly at the C6 level consistent with CT findings.

A 35-year-old male presented with a month-long history of dysphagia, severe headache, and posterior neck pain that radiated to the occiput. His medical history was significant for cervical surgeries, including an anterior cervical discectomy and fusion (ACDF) at C5/C6 one year and two months ago (Fig. 1). Shortly thereafter, the patient underwent revision surgery along with posterior plating from C5 to T1 due to osteomyelitis, worsening cervical deformity, and retropharyngeal abscess (Fig. 2). Initial workup included a noncontract computed tomography (CT), demonstrating increased gas density at C6 corpectomy site and post cricoid region (Fig. 3). A swallow study was obtained based on suspicion of a perforation and demonstrated extraluminal leakage of contrast posteriorly at the C6 level, consistent with initial CT (Fig. 4). Surgery included removal of the anterior and posterior hardware and esophageal repair. Although initially the esophageal injury was suspected to be related to the hardware, intraoperatively, it was found the instrumentation was not in communication with the esophagus. This confirmed the perforation was unrelated to direct injury from the cervical hardware construct, which was intact. The esophagus was repaired with a pectoralis flap. The postoperative course was uneventful, and the patient was discharged home after one week. The patient later died due to severe complications of substance abuse unrelated to the operation.

## Discussion

### Etiology

Esophageal perforation can occur intraoperatively or postoperatively following anterior cervical spinal surgery. Early postoperative injuries are often due to intraoperative manipulation [8], while delayed perforations, months to years later, are usually related to hardware failure or screw dislodgement. It is rare for perforation to occur over a year after successful fusion with intact instrumentation, making intraoperative injury less likely. These delayed perforations with intact hardware are hypothesized to result from pressure, microtrauma, and/or chronic friction on the posterior esophageal wall [9, 10]. Cervical osteophytes have also been suggested in esophageal injury after hyperextension trauma without prior surgery [11]. It is also important to recognize the initial reasons for an ACDF related to trauma versus related to nontraumatic causes (i.e. degenerative or infectious processes). It is difficult to pinpoint the exact cause of our patient given complicating factors including drug use, trauma, retropharyngeal abscess, and multiple cervical surgeries. Likely, minor injuries, infections, and reoperations could play a role in weakening the esophagus, leading to perforation despite intact hardware.

### Presentation and clinical diagnosis

Due to their delayed onset, these types of perforations can be difficult to recognize. Similar cases have been reported to present one to three years postoperatively, with one occurring as late as seven years [5, 8, 10, 12]. Common clinical signs include dysphagia, pain, crepitus, and fever and sometimes recurrent pneumonia and regurgitation [12]. In this case, the esophageal perforation was located at the C6 vertebral level. Pharyngoesophageal perforation (Zenker’s diverticulum) may present similary [12, 13] and should remain on the differential. The delayed presentation coupled with the indolent nature of symptoms emphasizes the importance of proper long-term follow-up in anterior cervical patients. As illustrated in a similar case [14] initial imaging such as X-rays and CTs can often be negative in this population, necessitating multiple studies before a diagnosis is confirmed. Dakwar *et al*. [12], demonstrated the utility of using multiple imaging modalities (barium swallow study, CT, and esophagoscopy) to improve diagnostic accuracy. A high index of suspicion is essential when evaluating patients with concerning symptoms and employ thorough imaging modalities including CT or magnetic resonance (MR) to assess the stability of the cervical hardware and/or fusion.

### Treatment and prognosis

The treatment generally consists of removing the spinal hardware, draining abscesses, and repairing the esophagus primarily with sutures, or a tissue flap. The sternocleidomastoid flap is the most commonly used flap, with additional support of using a pectoralis flap; primary sutures are typically used intraoperatively or in acute settings [7]. Caution should be used when considering primary closure as prior cases reported needing multiple attempts at closing the perforation with a primary closure [8]. Generally, patients who undergo surgical closure of the esophageal perforation have good outcomes. In the present case, the patient died shortly after due to severe complications from drug use unrelated to the esophageal and spinal operation. Previous studies have highlighted caution in using nonoperative treatment methods. Abscesses developed in 20%–25% of the patients treated, nonoperatively and the mortality rate was 18% [10, 15]. This emphasizes prompt recognition and appropriate surgical intervention is critical for better outcomes.

## Conclusion

Esophageal perforations can present over a year later following successful anterior cervical spinal surgery due to trauma without hardware failure. The esophageal perforation is likely due to microtrauma or pressure applied by the spine instrumentation over time. Given the delayed nature, once symptoms present it is important to consider this complication and surgically intervene if an esophageal perforation is found. Although rare, it can be life-threatening, and literature suggests a flap repair to the esophageal perforation may lead to better outcomes. Collaboration between neurosurgery and otolaryngology is important for assessment and plan to address this pathology.
